# Efflux pumps expression and its association with porin down-regulation and β-lactamase production among *Pseudomonas aeruginosa *causing bloodstream infections in Brazil

**DOI:** 10.1186/1471-2180-10-217

**Published:** 2010-08-12

**Authors:** Danilo E Xavier, Renata C Picão, Raquel Girardello, Lorena CC Fehlberg, Ana C Gales

**Affiliations:** 1 Division of Infectious Diseases, Universidade Federal de São Paulo, Rua Pedro de Toledo, 781, 04039-032, São Paulo, Brazil

## Abstract

**Background:**

Multi-drug efflux pumps have been increasingly recognized as a major component of resistance in *P. aeruginosa*. We have investigated the expression level of efflux systems among clinical isolates of *P. aeruginosa*, regardless of their antimicrobial susceptibility profile.

**Results:**

Aztreonam exhibited the highest in vitro activity against the *P. aeruginosa *isolates studied (64.4% susceptibility), whereas susceptibility rates of imipenem and meropenem were both 47.5%. The MexXY-OprM and MexAB-OprM efflux systems were overexpressed in 50.8% and 27.1% of isolates studied, respectively. Overexpression of the MexEF-OprN and MexCD-OprJ systems was not observed. AmpC β-lactamase was overexpressed in 11.9% of *P. aeruginosa *isolates. In addition, decreased *oprD *expression was also observed in 69.5% of the whole collection, and in 87.1% of the imipenem non-susceptible *P. aeruginosa *clinical isolates. The MBL-encoding genes *bla*_SPM-1 _and *bla*_IMP-1 _were detected in 23.7% and 1.7% *P. aeruginosa *isolates, respectively. The *bla*_GES-1 _was detected in 5.1% of the isolates, while *bla*_GES-5 _and *bla*_CTX-M-2 _were observed in 1.7% of the isolates evaluated. In the present study, we have observed that efflux systems represent an adjuvant mechanism for antimicrobial resistance.

**Conclusions:**

Efflux systems in association of distinct mechanisms such as the porin down-regulation, AmpC overproduction and secondary β-lactamases play also an important role in the multi-drug resistance phenotype among *P. aeruginosa *clinical isolates.

## Background

*Pseudomonas aeruginosa *is an aerobic gram-negative pathogen and a common etiologic agent of nosocomial infections, especially pneumonia, in seriously ill patients [1,2]. This species is intrinsically resistant to many antimicrobial agents and usually develop resistance to other antimicrobial agents during antimicrobial chemotherapy, further limiting the available therapeutic options [3].

Bacterial efflux systems capable of ejecting antimicrobials are mostly encoded by chromosomal genes and generally fall into five classes, the major facilitator superfamily (MFS), the ATP-binding cassette (ABC) family, the small multi-drug resistance (SMR) family, the multi-drug and toxic compound extrusion (MATE) family and the resistance-nodulation-division (RND) family [4]. The RND chromosomal systems are encoded by operons and are typically formed by three proteins, which are located in the inner membrane, periplasm and outer membrane of the bacterial cell [5].

Sequencing of *P. aeruginosa *genome revealed the presence of several RND efflux systems. Of those, MexAB-OprM, MexCD-OprJ, MexEF-OprN and MexXY-OprM are able to pump out multiple antipseudomonal compounds [1,4,6]. Studies with MexAB-OprM mutants demonstrated that this efflux system extrudes quinolones, aminoglycosides, macrolides, tetracycline, chloramphenicol, novobiocin, and most β-lactams but not imipenem [5]. The MexXY-OprM is able to eject cefepime, cefotaxime, levofloxacin, ciprofloxacin, amikacin, gentamicin, tobramycin, erythromycin, tetracycline and meropenem [5]. MexAB-OprM and MexXY-OprM are constitutively expressed and contribute to the intrinsic resistance phenotype of *P. aeruginosa*. However, when overexpressed, these efflux systems confer reduced susceptibility to different classes of antimicrobial agents [7,8]. Although the efflux systems MexCD-OprJ and MexEF-OprN are quiescent in wild type *P. aeruginosa*, their overexpression may also contribute to the acquired multi-drug resistance phenotype in mutant isolates [5].

Overexpression of efflux systems generally confers modest levels of antimicrobial resistance [9,10]. However, its association with other resistance determinants is frequently observed [11]. In Brazil, production of extended-spectrum β-lactamases (ESBL), such as CTX-M (cefotaximase) and GES (Guiana-extended spectrum), or metallo-β-lactamases (MBL) such as SPM (São Paulo Metallo-β-lactamase) and IMP (imipenemase) are the main mechanisms of acquired resistance to broad-spectrum β-lactams among *P. aeruginosa *clinical isolates [12]. The association of these β-lactamases with overexpression of efflux pumps and/or porin loss may lead to high level and/or co-resistance phenotypes [11]. For this reason, efflux pumps may seriously impact antimicrobial therapy in clinical settings. The aim of this study was to investigate the expression of efflux systems as well as its association with other resistance mechanisms, such as β-lactamase production and porin down-regulation, among *P. aeruginosa *clinical isolates.

## Results

### Bacterial isolates and antimicrobial susceptibility profile

Fifty-nine non-repetitive *P. aeruginosa *isolates were collected from bloodstream infections between June and December 2005. The majority of isolates was collected from patients hospitalized in intensive care units (64.4%), followed by the emergency room ward (28.8%) and pediatric oncology unit (6.8%). Aztreonam showed the highest susceptibility rate against the isolates studied (64.4%), followed by cefepime (49.2%), meropenem (47.2%), imipenem (47.2%), ceftazidime (44.1%), amikacin (40.7%), ciprofloxacin (35.6%) and gentamicin (32.2%, Table 1). Approximately 17% of the isolates (*n *= 10) were susceptible to all tested antimicrobial.

**Table 1:** The percentage of *P. aeruginosa *isolates that were non-susceptible to antimicrobials and demonstrated overexpression of efflux genes and *ampC *β-lactamase, coupled with *oprD *down-regulation.

Antimicrobial	Non-susceptible (*n *= 59)	% of isolates (*n*)			
		
		ABM+ (16)	XY+ (30)	AmpC+ (07)	OprD- (41)
Aztreonam	21 (35.6)	56.3 (09)	43.3 (13)	71.4 (05)	34.1 (14)

Imipenem	31 (52.5)	56.3 (09)	80.0 (24)	71.4 (05)	65.9 (27)

Meropenem	31 (52.5)	62.5 (10)	80.0 (24)	71.4 (05)	63.4 (26)

Cefepime	30 (50.8)	56.3 (09)	80.0 (24)	85.7 (06)	58.5 (24)

Ceftazidime	33 (55.9)	50.0 (08)	76.7 (23)	100 (07)	63.4 (26)

Amikacin	35 (59.3)	68.8 (11)	86.7 (26)	57.1 (04)	70.7 (29)

Gentamicin	40 (67.8)	75.0 (12)	86.7 (26)	57.1 (04)	65.9 (27)

Ciprofloxacin	38 (64.4)	81.3 (13)	86.7 (26)	85.7 (06)	63.4 (26)

The abbreviations ABM+, XY+ and AmpC+ designate MexAB-OprM, MexXY, and AmpC overexpression, respectively.

OprD -: OprD porin down-regulation.

### Pulsed Field Gel Electrophoresis

A total of 23 distinct PFGE patterns were detected among the 59 *P. aeruginosa *clinical isolates studied. Five *P. aeruginosa *isolates could not be typed by PFGE using SpeI. Although 38 isolates were clustered in six PFGE patterns, 16 isolates showed distinct PFGE patterns.

### Carbapenems hydrolysis and β-lactamases production

Carbapenem hydrolysis was detected in 15 *P. aeruginosa*, representing 25.4% of the whole collection and 48.4% of the imipenem-resistant isolates. These isolates had their carbapenemase activity inhibited by EDTA, and the presence of the MBL-encoding genes *bla*_SPM-1 _and *bla*_IMP_-like was confirmed by multiplex PCR, in 14 and 1 isolates, respectively. Among the SPM-producing *P. aeruginosa *studied, 13 showed the same PFGE pattern, whereas one isolate could not be typed using Spe I. ESBL-encoding genes were present in five isolates: *bla*_GES-1 _(*n *= 3), *bla*_GES-5 _(*n *= 1) and *bla*_CTX-M-2 _(*n *= 1). GES-type producers belonged to the same genotype, whereas CTX-M-2-producer showed a unique PFGE profile.

### Gene expression

The percentage of *P. aeruginosa *isolates that were non-susceptible to antimicrobials and demonstrated overexpression of efflux genes and *ampC*, coupled with *oprD *down-regulation is shown in Table 1. In addition, Table 2 shows the association of different resistance mechanisms identified, and antimicrobials MICs that were more frequently observed at each association (modal MIC).

**Table 2:** Association of resistance mechanisms identified among the *P. aeruginosa *isolates (*n *= 59) and the modal MICs for tested antimicrobials observed in each association.

Isolates and determinant of antimicrobial resistance (No. of isolates with OprD -)		**Modal MIC **^**a**^**(μg/ml)**							
		
		MER	IPM	ATM	CAZ	FEP	AMK	GEN	CIP
PAO1 reference strain	-	0.25	1	4	0.5	0.25	2	4	0.25-

No mechanisms of resistance identified	7 (0)	4	2	4-8^b^	4	2	8	4	16

XY+, MBL	7 (6)	>32	>32	8	256	>32	>256	>256	>32

XY+	7 (5)	16	8/16^b^	32	8/256^b^	>32	256	2/>256^b^	0.5/>32^b^

ABM+, XY+	5 (2)	0.25/8^b^	0.25/2^b^	16	8	4	256	2- >256^c^	32

ABM+, XY+, MBL	4 (3)	>32	>32	8	256	>32	>256	>256	>32

ABM+	3 (2)	0.5-16^b^	1	16	2-8^c^	4	4-32^c^	1-8^c^	0.25-8^c^

XY+, GES-1	3 (2)	8- >32^c^	8- >32^c^	8	128	>32	>256	256	16

ABM+, XY+, AmpC+	2 (2)	16/>32^b^	>32	8/32^b^	32/64^b^	16/32^b^	4/64^b^	1/8^b^	2/4^b^

ABM+, GES-5	1 (1)	>32	32	8	32	>32	128	128	32

ABM+, CTX-M2	1 (1)	4	1	>32	2	>32	128	256	16

XY+, AmpC+, MBL	2 (2)	32/>32^b^	>32	16/>32^b^	128/>256^b^	>32	>256	>256	>32

MBL	2 (2)	>32	>32	8	256	>32	>256	>256	32

AmpC+	3 (2)	1-8	2-16^c^	4-32^c^	16-256^c^	16	4	2	0.5-32^c^

OprD-	12 (12)	≤0.25	1-2	8	2	2	8	2	0.25

MER, meropenem; IPM, imipenem; ATM, aztreonam; CAZ, ceftazidime; FEP, cefepime; AMK, amikacin; GEN, gentamicin; CIP, ciprofloxacin.

The abbreviations XY+, ABM+, and AmpC+ designate MexXY, MexAB-OprM, and AmpC overexpression, respectively.

MBL, metallo-β-lactamase producer

OprD-, reduced expression of OprD porin.

^a^, Modal MIC is defined as the antimicrobial MICs that were more frequently observed at each association of resistance mechanisms.

^b^, two modal MICs observed;

^c ^MIC range when no modal MIC was observed.

The gene expression analysis showed that 50.8% (*n *= 30) and 27.1% (*n *= 16) of *P. aeruginosa *clinical isolates demonstrated increased *mexY *(from 2.2- to 41.0-fold) and *mexB *(from 2.1- to 10.0-fold) transcription mRNA levels, respectively, compared to those of PAO1. In addition, 11 *P. aeruginosa *isolates (18.6%) showed overexpression of both *mexB *and *mexY *efflux genes. Overexpression of MexCD-OprJ and MexEF-OprN were not observed among the clinical isolates of *P. aeruginosa *evaluated in this study. Overall, 69.5% and 11.9% of *P. aeruginosa *clinical isolates studied showed decreased *oprD *expression (from 0.1- to 0.7-fold compared to PAO1), and overexpression of *ampC *(from 14- to 402-fold compared to PAO1), respectively. None of the investigated resistance determinants was identified in 11.8% of clinical isolates (*n *= 7, Table 2).

Among the isolates overexpressing the *mexY *efflux gene, 86.7% were not susceptible to amikacin, gentamicin and ciprofloxacin. Cefepime non-susceptibility was observed in 80% of isolates overexpressing *mexY*. Of those, 79.2% also presented reduced *oprD *transcription, 54.2% were MBL-producers, 12.5% produced the ESBL GES-1, and 16.7% showed increased *ampC *transcriptional levels (data not shown). Among the cefepime non-susceptible isolates that did not show *mexY *overexpression, 33.3% produced SPM-1, 33.3% overexpressed *ampC*, 16.7% produced the ESBL CTX-M-2, and 16.7% produced GES-5, an ESBL with carbapenemase activity.

Meropenem non-susceptibility was observed among 62.5% of isolates overexpressing *mexB *(from 2.1- to 5.5-fold higher than PAO1). Of those, 90.0% showed decreased *oprD *expression, 40.0% were MBL producers, 20.0% overexpressed *ampC *and 10.0% were GES-5 producers (data not shown). As expected, all meropenem-susceptible isolates that overexpressed *mexB*, presented normal expression of both *ampC *and *oprD *when compared to that of PAO1. Higher percentage of *mexB *overexpression was observed among isolates that were also not susceptible to cefepime, amikacin, gentamicin and ciprofloxacin. Of note, 85.7% and 28.6% of SPM-producing *P. aeruginosa *showed increased transcriptional levels of *mexY *and *mexB*, respectively.

It is worth to mention that MexAB-OprM and/or MexXY-OprM overexpression was observed among isolates that were susceptible to most antimicrobials tested. This finding was expected since efflux pump overexpression in *P. aeruginosa *usually confers modest increase in the MICs of antimicrobial agents that are ejected by these systems.

## Discussion and Conclusions

*P. aeruginosa *is the fifth most frequent pathogen of bloodstream infections and the first one causing pneumonia in Latin America according to the SENTRY Antimicrobial Surveillance Program [13]. In the last decades, the emergency of multi-drug resistant *P. aeruginosa *has been observed worldwide. Some of antimicrobial agents have become less effective against these organisms reducing the available therapeutic options for treatment of these infections.

In this study 52.5% of the *P. aeruginosa *isolates studied were resistant to carbapenems. Our findings are in accordance of previous studies that showed high rates of antimicrobial resistance, including carbapenems, among *P. aeruginosa *clinical isolates collected from Brazilian institutions [14]. The genetic diversity observed among the *P. aeruginosa *isolates studied indicates that spread of clones and emergency of distinct genotypes have occurred in our hospital. The high rate of carbapenem resistance can be partially explained by the spread of an endemic SPM-producing clone. It also justifies the susceptibility rate to aztreonam since MBL producers are not able to hydrolyze this antimicrobial agent. This finding corroborates with those previously reported that described a single SPM producer clone spread out in the Brazilian territory [15].

The overexpression of efflux systems may impact on clinical outcome of *P. aeruginosa *infections since they are capable of pumping out many classes of antimicrobial agents used for treatment of these infections [16]. However, it has not been clearly established the correlation between increase in the transcriptional level of an efflux-encoding gene and antimicrobial resistance leading to possible therapeutic failure [17].

In the present study, we have evaluated the transcriptional levels of four efflux-encoding genes as well as *ampC *and *oprD *among 59 *P. aeruginosa *clinical isolates. This collection represents the total number of patients with bloodstream infection due to *P. aeruginosa *in a six-month period in Hospital São Paulo, Brazil. We also aimed to evaluate the frequency of isolates presenting different mechanisms of β-lactam resistance and their association.

The overexpression of the MexAB-OprM and MexXY-OprM efflux systems were more frequent among antimicrobial resistant *P. aeruginosa *isolates. Since MexAB-OprM and MexXY-OprM are constitutively expressed in wild type *P. aeruginosa *isolates, the antimicrobial policy in use in each individual institution may interfere with the selection of the most overexpressed efflux system. Aminoglycosides are important substrates of MexXY-OprM and might have exerted a role in selecting *P. aeruginosa *that overexpressed this system [18]. The expression of MexXY-OprM is inducible, while expression of MexAB-OprM is not [5]. In our institution, the prescription of aminoglycosides is not controlled and these antimicrobial agents usually are prescribed in combination for treatment of *P. aeruginosa *infections. These facts could in part justify why MexXY-OprM was the most frequent overexpressed efflux system, since *mexXY *expression may be induced by these antimicrobial class [19]. Interestingly, the overexpression of MexXY-OprM was observed in all MBL-producing isolates.

We did not notice a strict correlation between antimicrobial resistance and efflux genes overexpression. However, efflux overexpressing isolates often presented higher antimicrobial MICs than did PAO1 and those isolates in which no antimicrobial resistance determinant was found.

Our findings clearly demonstrate that β-lactamase production increase antimicrobial MICs more efficiently than do efflux overexpression or porin down-regulation alone. However, these chromosomal resistance mechanisms were frequently present among acquired β-lactamase producers. These findings suggest that efflux overexpression and porin down-regulation may favor the bacterial survival under selective pressure, increasing its chance to acquire further resistance determinants.

In the present study we have observed that efflux pump overexpression do not appear to be the main mechanism of drug resistance among the studied clinical isolates of *P. aeruginosa*, but represents an adjuvant mechanism for antimicrobial resistance. The association of distinct mechanisms such as the porin down-regulation and AmpC overproduction play also an important role in the multi-drug resistance phenotype among *P. aeruginosa *clinical isolates studied. In addition, our findings indicate that spread of clones and emergency of distinct genotypes have occurred in our institution and implementation of control measures is extremely necessary to modify this scenario.

## Methods

### Bacterial isolates and antimicrobial susceptibility testing

With the approval of the local Ethics in Research committee (Comitê de Ética em Pesquisa Hospital São Paulo, protocol number: CEP0398/07), a total of 59 clinical isolates of *P. aeruginosa *were evaluated, regardless of their antimicrobial susceptibility profile. These isolates were consecutively collected between June and December 2005 from blood culture of patients hospitalized at Hospital São Paulo, a tertiary teaching hospital located in São Paulo, Brazil. Only a single bacterial isolate per patient was evaluated. MICs for ceftazidime, cefepime, aztreonam, imipenem, meropenem, gentamicin, amikacin and ciprofloxacin were determined by agar dilution and interpreted according to Clinical Laboratory Standards Institute [20,21]. *P. aeruginosa *ATCC 27853 and *Escherichia coli *ATCC 25922 strains were used as quality control strains.

### Pulsed Field Gel Electrophoresis

Genomic DNA of isolates was prepared in agarose blocks and digested with the restriction enzyme SpeI (New England, Beverly, MA). Electrophoresis was performed on CHEF-DR III (BioRad, Richmond, CA), with the following conditions: 0.5 × TBE, 1% agarose, 13°C, 200 V, for 24 h with switch time ramped from 5 to 90 s. The band patterns were interpreted as previously recommended [22].

### Screening for carbapenemase producers and detection of β-lactamases-encoding genes

Investigation of carbapenemase activity in crude extracts was performed by UV spectrophotometric assays. Briefly, a full 10 μl loop of the test organism was inoculated into 500 μl of phosphate buffer 100 mM (pH 7.0) and disrupted by sonication. The cells were removed by centrifugation and the supernatants were used for further experiments. Protein quantification in the crude extracts was performed using the Bradford stain. Hydrolytic activity of crude extracts was determined against 100 μM imipenem and 100 μM meropenem in 100 mM phosphate buffer (pH 7.0). Measurements were carried out at a 297 nm wavelength. Positive control included SPM-1-producing *P. aeruginosa *48-1997A [23]. Carbapenem hydrolysis inhibition was performed by incubating the crude extract with 25 mM EDTA during 15 min, previously to the assay with imipenem and meropenem. Detection MBL-encoding genes was performed for all carbapenem-resistant isolates by multiplex PCR, as previously described [24]. The presence of ESBL-encoding genes *bla*_TEM_, *bla*_SHV_, *bla*_CTX-M_, *bla*_GES_, *bla*_VEB _and *bla*_PER _was investigated by PCR, as previously reported [12,25].

### Quantitative RT-PCR (RT-qPCR)

Transcriptional levels of *mexB*, *mexD*, *mexF*, *mexY*, *ampC *and *oprD *were determined with Mastercycler Realplex^2 ^(Eppendorf, Hamburg, Germany). In brief, total RNA was extracted using the RNase Mini Kit, following the manufacturer recommendations (Qiagen, Hilden, Germany). Five micrograms of total RNA was submitted to cDNA synthesis using High Capacity cDNA Archive Kit (Applied Biosystems, Foster City, USA). Quantitative RT-PCR was performed with Platinum SYBR Green Supermix (Invitrogen, Carlsbad, USA), using specific primers for *mexB*, *mexD*, *mexF*, *mexY*, *ampC *and *oprD *as previously described [26-29] or designed for this study using the GeneFisher online software http://bibiserv.techfak.uni-bielefeld.de/genefisher/old.html (Table 3). Amplification was carried out in triplicate from cDNA preparations. To assure that specific amplification had occurred, melting curves of each amplicon was assessed and compared to that Tm obtained when using PAO1 DNA total was tested as template. A gene encoding the ribosomal protein *rpsL *was used as a reference gene for normalizing the transcriptional levels of target genes. Transcription data were analyzed with the Q-Gene software [30]. According to previous studies [31] the efflux systems MexAB-OprM, MexCD-OprJ, MexEF-OprN, and MexXY were considered overexpressed when the transcriptional levels of *mexB*, *mexC*, *mexE*, and *mexY *were at least 2, 100, 100, and 4 fold higher than those of the wild-type reference strain PAO1, respectively. Reduced *oprD *expression and overexpression of *ampC *were considered relevant when their transcriptional levels were ≤70% and ≥10-fold, respectively, compared to that of the PAO1 reference strain [10,32].

**Table 3:** Primers used in this study for access the relative gene expression by RT-qPCR

Genes	Primers	Sequences (5'-3')	Amplicon size (bp)	References
*mexB*	mexB-F	GTGTTCGGCTCGCAGTACTC	244	[26]
			
	mexB-R	AACCGTCGGGATTGACCTTG		

*mexD*	mexD-F	CGAGCGCTATTCGCTGC	165	This study
			
	mexD-R	GGCAGTTGCACGTCGA		

*mexF*	mexF-F	CGCCTGGTCACCGAGGAAGAGT	255	[27]
			
	mexF-R	TAGTCCATGGCTTGCGGGAAGC		

*mexY*	mexY-F	CCGCTACAACGGCTATCCCT	250	[26]
			
	mexY-R	AGCGGGATCGACCAGCTTTC		

*oprD*	oprD-F	TCCGCAGGTAGCACTCAGTTC	191	[28]
			
	oprD-R	AAGCCGGATTCATAGGTGGTG		

*ampC*	ampC-F	CTGTTCGAGATCGGCTC	166	This study
			
	ampC-R	CGGTATAGGTCGCGAG		

*rpsL*	rpsL-F	GCAAGCGCATGGTCGACAAGA	201	[29]
			
	rpsL-R	CGCTGTGCTCTTGCAGGTTGTGA		

## Competing interests

The authors declare that they have no competing interests.

## Authors' contributions

All authors had equal contribution in preparing this article. DEX drafted the first manuscript of this article based on his MSc thesis, which was supervised by RCP and ACG. RG was involved in the determination of antimicrobial susceptible profile. LCCF carried out the molecular typing and was involved in the determination of the gene transcriptional level. All authors read and approved the final manuscript.

## Funding

This work was financially supported by Fundação de Amparo à Pesquisa do Estado de São Paulo (FAPESP - 2006/01716-8), by Coordenação de Aperfeiçoamento de Pessoal de Nível Superior (CAPES) that conceded a grant to DEX and Conselho Nacional de Desenvolvimento Científico e Tecnológico (CNPq) that provides a researcher grant to ACG. (307714/2006-3).
